# Genetic Contribution of Synapse-Associated Protein 97 to Orbitofrontal–Striatal–Thalamic Circuitry Connectivity Changes in First-Episode Schizophrenia

**DOI:** 10.3389/fpsyt.2021.691007

**Published:** 2021-07-19

**Authors:** Xusan Xu, Shucun Luo, Xia Wen, Xiaoxia Wang, Jingwen Yin, Xudong Luo, Bin He, Chunmei Liang, Susu Xiong, Dongjian Zhu, Jiawu Fu, Dong Lv, Zhun Dai, Juda Lin, You Li, Zhixiong Lin, Wubiao Chen, Zebin Luo, Yajun Wang, Guoda Ma

**Affiliations:** ^1^Institute of Neurology, Affiliated Hospital of Guangdong Medical University, Zhanjiang, China; ^2^Maternal and Children's Health Research Institute, Shunde Women and Children's Hospital, Guangdong Medical University, Foshan, China; ^3^Department of Radiology, Affiliated Hospital of Guangdong Medical University, Zhanjiang, China; ^4^Department of Psychiatry, Affiliated Hospital of Guangdong Medical University, Zhanjiang, China

**Keywords:** schizophrenia, SAP97, rs3915512, resting state functional connectivity, deterministic tractography

## Abstract

Functional and structural disturbances in the orbitofrontal–striatal–thalamic circuitry are thought to be associated with mental symptoms and neurocognitive impairments in schizophrenia. This study tested whether synapse-associated protein 97 (SAP97), a reasonable candidate gene for schizophrenia, is related to orbitofrontal–striatal–thalamic connection changes in first-episode schizophrenia (FES) patients and the clinical performance of schizophrenic patients by affecting this integrity. Fifty-two FES patients and 52 matched healthy controls were recruited. All subjects underwent genotyping via the improved multiplex ligation detection reaction technique and scanning with magnetic resonance imaging (MRI) to provide orbitofrontal–striatal–thalamic functional and structural imaging data. A two-way analysis of covariance model was employed to examine abnormal brain connectivities, and Spearman correlations were applied to estimate the relationships between brain connectivity and clinical manifestations. In the FES group, those with the SAP97 rs3915512 TT genotype showed lower structural and functional connectivity than A allele carriers between the orbitofrontal gyrus and striatum/thalamus. In the FES group, negative correlations were found between resting-state functional connectivity (RSFC) in the orbitofrontal gyrus and thalamus, and positive symptoms between structural connections in the orbitofrontal gyrus and striatum and cognitive functions, and positive correlations were suggested between RSFC in the orbitofrontal gyrus and thalamus and negative symptoms. Our findings suggested that the SAP97 rs3915512 polymorphism may be involved in mental symptoms and cognitive dysfunction in FES patients by influencing structural and functional connectivity of the orbitofrontal–striatal and orbitofrontal–thalamic regions.

## Introduction

Current thinking suggests that schizophrenia relates to disturbances in neural connectivity (1). Abnormal connectivity in the orbitofrontal–striatal–thalamic circuitry has been closely linked to neuropsychiatric symptoms and neurocognitive impairments in schizophrenic patients (2). Genetic factors are thought to be the main risk factors for schizophrenia (3). Further work is needed to determine whether connectivity changes are related to genetic risk for schizophrenia.

Reduced mRNA (4) and protein expression (5) of synapse-associated protein 97 (SAP97) in schizophrenic patients indicated that the SAP97 gene may play a potential role in schizophrenia. Indirect interactions with D4 dopamine receptors can enhance α-amino-3-hydroxy-5-methyl-4-isoxazole propionic acid receptor (AMPAR) responses in the low activity state of prefrontal neurons (6), suggesting the possibility that SAP97 may work on neural connectivity. Our previous study indicated that SAP97 gene polymorphisms (rs3915512 and other polymorphisms) were related to cognitive dysfunction and negative symptoms of schizophrenia (7, 8). Moreover, the T>A variation of SAP97 rs3915512 was found to truncate the scaffold protein encoded by SAP97 (4). Therefore, we speculate that SAP97 may be a risk gene that can cause disrupted neural connectivity in schizophrenia and that SAP97 rs3915512 is involved.

Our primary goal was to evaluate whether structural and functional connectivity have significant disease × SAP97 interactive effects in the orbitofrontal–striatal–thalamic circuitry. Then, we analyzed the relationship between abnormal connectivities and clinical characteristics. First-episode schizophrenia (FES) patients were recruited in this study to avoid long-term disease processes or treatment effects.

## Materials and Methods

### Subjects and Genotyping

This study enrolled 104 participants, including 52 FES patients (30 males and 22 females) and 52 healthy controls (HCs) (23 males and 29 females). As described previously (8), subjects with substance abuse, organic brain damage, metabolic diseases, or any other severe physical diseases were removed from this study. More details on data collection in these participants are presented in the Supplementary Material. The demographic characterization of the involved participants is shown in Table 1. This research was approved by the ethics committee of the Affiliated Hospital of Guangdong Medical University. All involved individuals gave written informed consent. As described in previous articles (7), the improved multiplex ligation detection reaction technique (Genesky Biotech, Shanghai, China) was used in SAP97 rs3915512 genotyping.

**Table 1:** Demographic, genotype, and clinical details in HC and FES groups.

**Group**	**HC**	**FES**			***p*-value**
N	52	52			
HWE	0.484	0.324			
Age (years)	29.17 ± 8.48	27.29 ± 8.21		*F* = 0.73	0.252
Male/female	23/29	30/22		χ^2^ = 1.89	0.107
Education (years)	11.62 ± 2.61	10.60 ± 2.70		*F* = 0.50	0.053
TT	30	28			
TA/AA	19/3	22/2		χ^2^ = 0.16	0.693
MAF	0.24	0.25			
T	79	78			
A	25	26		χ^2^ = 0.03	0.872
PANSS		TT(*n* = 26)	TA+AA(*n* = 24)		
Total score		80.62 ± 23.24	78.67 ± 18.40		0.745
Positive score		25.54 ± 9.24	22.29 ± 9.34		0.223
Negative score		17.62 ± 13.16	20.83 ± 14.72		0.418
Pathological score		37.46 ± 10.98	35.54 ± 8.22		0.490
BACS		TT (*n* = 17)	TA+AA (*n* = 15)		
Working memory	–	23.47 ± 5.04	19.38 ± 6.28		0.067
Semantics fluency	–	33.77 ± 9.89	30.92 ± 6.16		0.393
Letter fluency	–	12.33 ± 5.23	12.08 ± 2.97		0.884
Verbal memory	–	37.27 ± 8.75	31.78 ± 8.79		0.180
Motor speed	–	45.35 ± 14.82	43.10 ± 14.06		0.701
Reasoning and problem solving	–	15.58 ± 5.18	12.64 ± 4.90		0.177
Attention and processing speed	–	33.46 ± 9.79	32.58 ± 9.37		0.821

*HWE, Hardy–Weinberg equilibrium; MAF, minor allele frequency. Some of these data come from our previous publication (8)*.

### Neuroimaging Acquisition Methods and Data Processing

T1-weighted data, functional MRI (fMRI) data, and diffusion tensor imaging (DTI) data were collected in a 3.0-T GE Discovery MR750 scanner (GE Healthcare Systems, Milwaukee, WI, USA) system. Only the main steps are described below, and complete details of the Montreal Neurological Institute (MNI) coordinates of seeds, data acquisition, and analysis are presented in the Supplementary Material.

The analysis of the fMRI data was performed by resting-state fMRI (DPARSF_V2.3, Cognitive and Brain Diseases Center of Hangzhou Normal University). For the dataset of each subject, the main steps for fMRI data processing included removal of the first 10 time points, slice timing correction, head motion correction, normalization to the MNI template space, spatial smoothing, and seed-based region of interest (ROI)-wise functional connectivity analysis.

The analysis of the DTI data was performed by the software pipeline toolbox for analyzing brain diffusion images (PANDA, State Key Laboratory of Cognitive Neuroscience and Learning and IDG/McGovern Institute for Brain Research, Beijing Normal University) (9). Major steps for DTI data processing included extracting the brain, correcting eddy current/motion, averaging acquisitions, and performing deterministic tracking.

### Statistical Analyses

The TA and AA genotype groups were merged for analysis because of the low number of individuals with the AA genotype. All the statistical analyses in this study were estimated via SPSS 21.0 software. With the resting-state functional connectivity (RSFC) value, fiber number, fractional anisotropy (FA) value, and fiber length as independent variables, and educational year, age, and sex as covariates, 2 × 2 analysis of covariance (ANCOVA) was used to examine the interactive effect of disease and genotype, and *post hoc t*-test analysis was applied to evaluate the details of the interactive effects. Correction for multiple comparisons was carried out by Bonferroni correction at *p* < 0.05.

### Correlation Analysis Between Brain Alterations and Clinical Performance Scores

We used Spearman correlation to estimate the relationships between brain activity (RSFC value, fiber number, FA value, and fiber length) and clinical performance [the Positive and Negative Symptom Scale (PANSS) and the Brief Assessment of Cognition in Schizophrenia (BACS) scale scores] in each patient group. After Bonferroni correction, *p* < 0.05 was considered statistically significant.

## Results

In terms of gender, age, and education, the FES group matched well-with the HC group (*p* > 0.05) (Table 1). There were no significant differences between the patients with the TT genotype and A allele carriers in the FES group in PANSS scores and BACS scores (*p* > 0.05) (Table 1).

**Figure 1:**
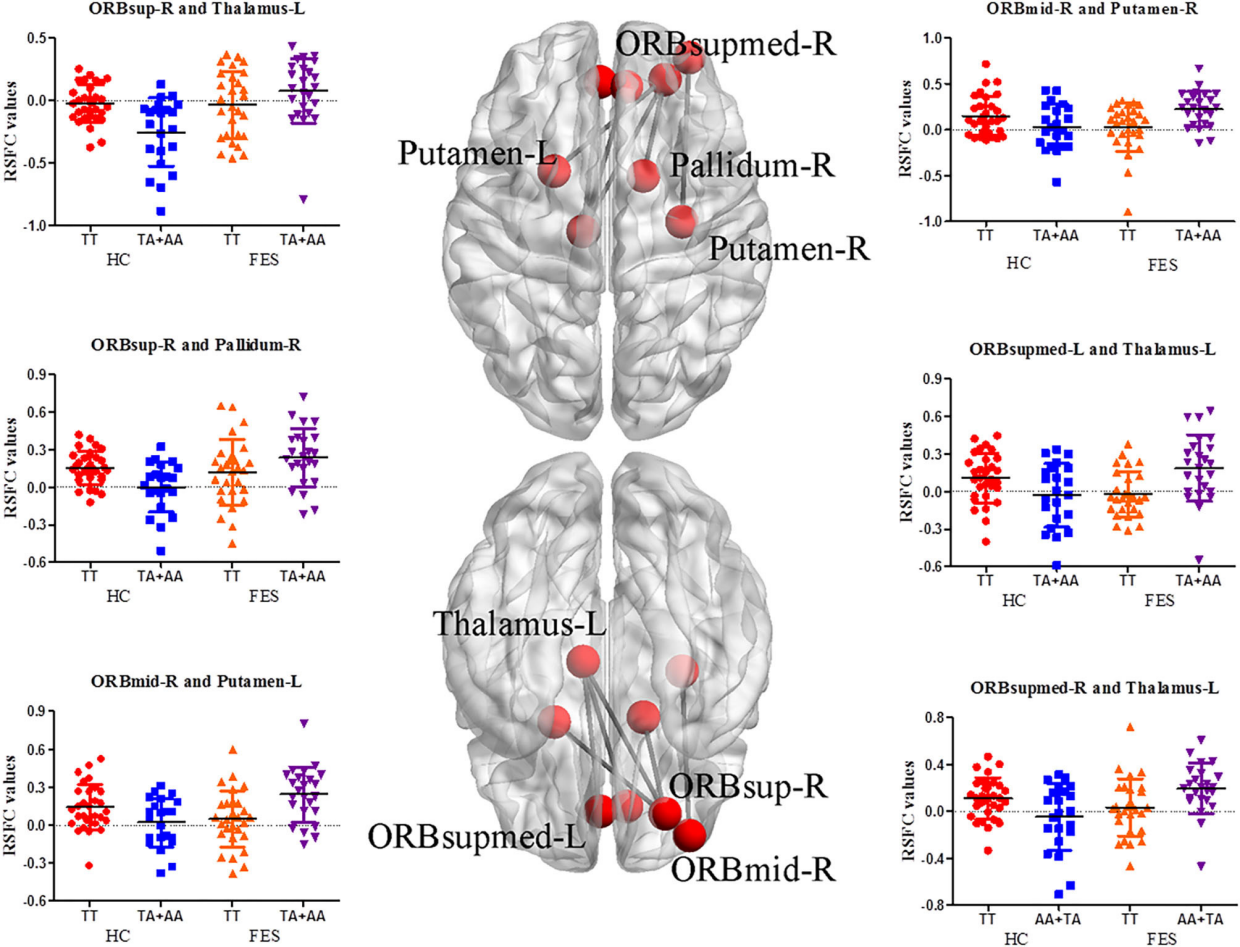
RSFC analysis between orbitofrontal, striatal, and thalamic regions in the FES group. On both sides of the figure are scatter plots with significantly altered functional connectivity between brain areas. The nodes on the brain model indicate the brain regions showing interactions, and the edges represent the mean Z-values between brain areas (edited by BrainNet Viewer software, National Key Laboratory of Cognitive Neuroscience and Learning, Beijing Normal University, China). R, right; L, left; ORBsup, superior frontal gyrus, orbital part; ORBmid, middle frontal gyrus, orbital part; ORBsupmed, superior frontal gyrus, medial orbital part.

As shown in Supplementary Table 2 and Figure 1, significant interactive effects between diagnosis (FES and HC) and SAP97 genotype (TT and TA+AA) were observed in the RSFC of the right superior frontal gyrus, orbital part with the left thalamus and right pallidum (*p* = 3.92E-04 and 0.001, respectively); RSFC of the right middle frontal gyrus, orbital part with the left and right putamen (*p* = 2.83E-04 and 0.001, respectively); and RSFC of the left thalamus with the left and right superior frontal gyrus, medial orbital part (*p* = 1.12E-04 and 3.76E-04, respectively). In the *post hoc* analysis of genotype, the TT genotype showed lower RSFC between the right middle frontal gyrus, orbital part and the left and right putamen (*p* = 0.001 and 0.004, respectively), and between the left thalamus and the left and right superior frontal gyrus, medial orbital part (*p* = 0.001 and 0.008, respectively) (Supplementary Table 2).

Significant interactive effects on FA values between the right middle frontal gyrus, orbital part and right pallidum (*p* = 0.006); on fiber numbers between the left middle frontal gyrus, orbital part and left thalamus and between the right middle frontal gyrus, orbital part and right putamen (*p* = 0.002 and 0.003, respectively); and on fiber length between the left superior frontal gyrus, orbital part and left caudate and between the right middle frontal gyrus, orbital part and right pallidum (*p* = 0.005 and 0.008, respectively) (Table 2). Patients with the TT genotype showed lower fiber number between the left middle frontal gyrus, orbital part and left thalamus (*p* = 0.003) in the *post hoc* analysis of genotype (Table 2).

**Table 2:** Interactive effects and *post hoc* analysis of FA values, fiber number, and fiber length between the SAP97 rs3915512 genotype and disease.

**AAL**			**HC**		**FES**		**Interactive effect**		***post hoc*** **analysis of genotype in FES**	
			**TT**	**TA+AA**	**TT**	**TA+AA**	***F***	***p***	***F***	***P***
ORBsup-L	Caudate-L	LEN (mm)	30.30 ± 17.32	19.81 ± 12.90	27.66 ± 18.30	36.09 ± 14.48	8.09	0.005	3.34	0.071
ORBmid-L	Thalamus-L	FN	25.24 ± 31.92	11.19 ± 12.11	15.81 ± 26.20	47.54 ± 66.22	9.77	0.002	9.39	**0.003**
ORBmid-R	Pallidum-R	FA	0.31 ± 0.19	0.16 ± 0.21	0.17 ± 0.21	0.27 ± 0.21	7.91	0.006	2.55	0.114
ORBmid-R	Putamen-R	FN	34.93 ± 31.39	7.48 ± 10.00	20.96 ± 26.60	35.79 ± 44.71	9.06	0.003	1.93	0.168
ORBmid-R	Pallidum-R	LEN (mm)	40.06 ± 25.77	22.15 ± 29.62	22.13 ± 27.30	35.55 ± 28.75	7.35	0.008	2.80	0.097

*AAL, anatomical automatic labeling. Values are the mean ± SD; 2 × 2 ANCOVA, p < 0.00833. The bold values in the post hoc analysis survived Bonferroni correction (p < 0.0125)*.

*R, right; L, left; ORBsup, superior frontal gyrus, orbital part; ORBmid, middle frontal gyrus, orbital part; LEN, fiber length; FN, fiber number*.

**Figure 2:**
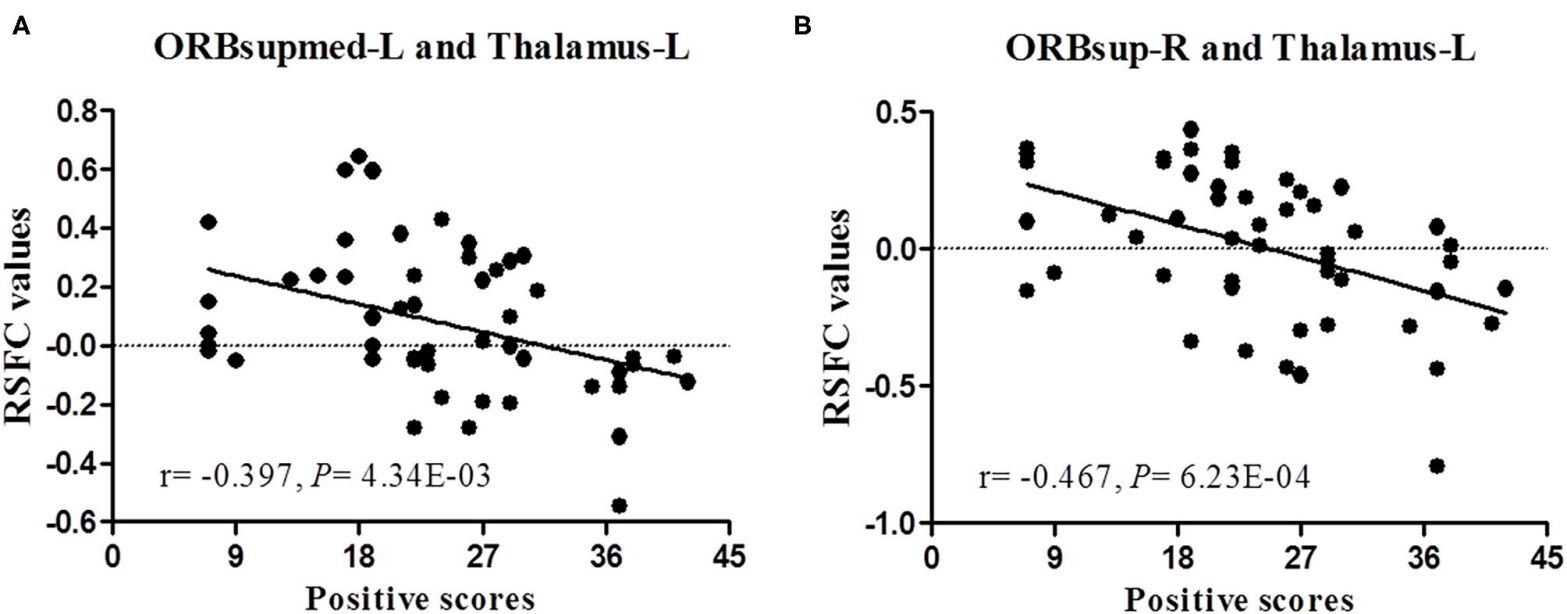
Correlation analysis between RSFC and positive symptom scores of patients with schizophrenia. **(A,B)** represent a significant negative correlation between RSFC and positive symptom scores after Bonferroni correction (*p* < 0.005).

Spearman correlations revealed significant negative correlations in the FES patients between PANSS positive symptom scores and RSFC between the left superior frontal gyrus, medial orbital part and left thalamus (*r* = −0.397, *p* = 4.34E-03), between the right superior frontal gyrus, medial orbital part and left thalamus (*r* = −0.389, *p* = 0.005), and between the right superior frontal gyrus, orbital part and left thalamus (*r* = −0.467, *p* = 6.23E-04); between FA in the right middle frontal gyrus, orbital part and right pallidum and BACS letter fluency scores (*r* = −0.385, *p* = 0.047); between FA in the right middle frontal gyrus, orbital part and right pallidum and BACS verbal memory scores (*r* = −0.458, *p* = 0.042); between fiber length between the right middle frontal gyrus, orbital part and right pallidum and BACS fluency scores (*r* = −0.387, *p* = 0.046); and between fiber length between the right middle frontal gyrus, orbital part and right pallidum and BACS verbal memory scores (*r* = −0.456, *p* = 0.043). Moreover, significant positive correlations were found between PANSS negative symptom scores and RSFC of the right superior frontal gyrus, medial orbital part and left thalamus (*r* = 0.281, *p* = 0.048) and right superior frontal gyrus, orbital part and left thalamus (*r* = 0.309, *p* = 0.029). Only the correlations between RSFC of the left superior frontal gyrus, medial orbital part and left thalamus and the right superior frontal gyrus, orbital part and left thalamus and PANSS positive symptom scores survived the multiple comparison correction (*p* < 0.005) (Table 3, Figure 2).

**Table 3:** Correlations between brain changes and clinical symptom scores.

**Parameter**	**Brain region**			***r*-value**	***p***
			**PANSS score**		
RSFC	ORBsupmed-L	Thalamus-L	Positive score	−0.397	**4.34E-03**
	ORBsupmed-R	Thalamus-L	Positive score	−0.389	0.005
	ORBsupmed-R	Thalamus-L	Negative score	0.281	0.048
	ORBsup-R	Thalamus-L	Positive score	−0.467	**6.23E-04**
	ORBsup-R	Thalamus-L	Negative score	0.309	0.029
			BACS score		
FA	ORBmid-R	Pallidum-R	Letter fluency	−0.385	0.047
	ORBmid-R	Pallidum-R	Verbal memory	−0.458	0.042
LEN(mm)	ORBmid-R	Pallidum-R	Letter fluency	−0.387	0.046
	ORBmid-R	Pallidum-R	Verbal memory	−0.456	0.043

*LEN, fiber length; R, right; L, left; ORBsup, superior frontal gyrus, orbital part; ORBmid, middle frontal gyrus, orbital part; ORBsupmed, superior frontal gyrus, medial orbital part*.

*r-value: correlation coefficient with bold values indicating statistical significance after Bonferroni correction (p < 0.005)*.

## Discussion

Accumulating evidence suggests that orbitofrontal gyrus abnormalities are involved in psychiatric symptoms and cognitive deficits associated with schizophrenia (10). The main outputs of the orbital frontal gyrus connect to the striatum, thalamus, and other parts of the prefrontal cortex (11). Several lesion studies have demonstrated key roles for the orbitofrontal–striatal and orbitofrontal–thalamic connections in the occurrence and development of psychiatric symptoms and cognitive disorders in schizophrenia (12, 13). This study first found significant genotype × disease interactive effects on RSFC between the orbitofrontal gyrus and striatal (pallidum and putamen) regions and between the orbitofrontal gyrus and thalamic regions in the FES patients (Supplementary Table 2). Further exploration showed that there were also disturbed structural links between these regional brain areas (Table 3). Therefore, SAP97 may be a candidate gene that can result in dysconnectivity between the orbitofrontal–striatal and orbitofrontal–thalamic regions in schizophrenia. SAP97 rs3915512, which can affect the structure of the SAP97-encoded protein (4), may be involved in these processes.

Several studies have reported abnormal orbitofrontal–striatal activity during performance of various cognitive tasks in those with schizophrenia (14, 15). Here, we reduplicated previous observations, and our data showed that the SAP97 rs3915512 TT genotype had lower functional connectivity (RSFC value) than A allele carriers between the orbitofrontal gyrus and striatum (right pallidum and bilateral putamen) in the FES group (Supplementary Table 2). In addition, decreased structural connectivity (FA value, fiber number, and fiber length) was found between the orbitofrontal gyrus and striatum (right pallidum, right putamen, and left caudate) (Table 3). The caudate, as a cognitive region, interacts with orbitofrontal regions involved in executive function processes (supporting reasoning, behavioral planning, and shifts in response strategy) (16). Clarke et al. found that orbitofrontal depletion can upregulate caudate dopamine and alter behavior (14). Combining previous reports and our results suggests that increased connectivity in the FES with the risk allele group may be a compensatory mechanism for the relatively inefficient connectivity of the relevant brain regions to reach near-normal performance (17). Consistent with a recent task-independent fMRI study, we also found abnormal orbitofrontal–putamen functional connectivity in schizophrenia but failed to associate connectivity with the severity of negative symptoms in that study (18). Few previous reports have mentioned abnormal orbitofrontal–pallidum connectivity in schizophrenia. Pallidotomy or pallidum stimulation was reported to significantly change several types of cognition (letter-cued fluency and category-cued fluency) (19). In the present study, structural connectivity of the orbitofrontal–pallidum was increased in the FES with the risk allele group, and this negatively correlated with cognitive scores (letter fluency and verbal memory). In short, the SAP97 rs3915512 polymorphism may affect cognition in schizophrenic patients by regulating orbitofrontal–striatal brain connectivity.

The thalamus has widespread connections with the cortex and plays a crucial role in cortico-cortical communication, emotion, and cognition (20). Altered connectivity between the orbitofrontal and thalamus has been frequently reported in schizophrenia (20, 21). Our research also found this abnormal connection, and the FES with the risk allele group had greater thalamic connectivity with the orbitofrontal region. Abnormal functional and structural connectivity between the orbitofrontal gyrus and thalamus have been proposed to underlie positive and negative symptoms of schizophrenia (21, 22). In this study, we also observed that the magnitude of connectivity between the orbitofrontal and thalamus was negatively correlated with positive symptoms and positively correlated with negative symptoms.

A limitation was the relatively small number of available genotyped patients with schizophrenia, although it seems safe to conclude that our data were sufficient to be generalized to a portion of those with schizophrenia in the Chinese Han population (the minor allele frequency of 3915512 in the population in southern China reported by the 1,000 Genomes project and HapMap is 0.28, while it was 0.24 in our cohort). Another limitation of this study was the use of a small number of ROIs to represent the whole orbitofrontal, striatal, and thalamic regions to detect connectivity relationships. These brain regions contain many subnuclei with distinct connections to specific brain regions that play specific functions. However, when exploring specific subnuclei, Anticevic et al. found similar connectivity patterns (23).

## Conclusions

In summary, our results replicated functional and structural dysconnectivity in the orbitofrontal–striatal and orbitofrontal–thalamic regions in FES and further revealed that the SAP97 rs3915512 polymorphism was involved in this process. Patients with the SAP97 risk allele appear to have more severe cognitive disorders and negative symptoms but milder positive symptoms, suggesting that compensatory increases in RSFC, FA value, fiber length, and fiber number in these regions may contribute to dysfunction. Future studies should include longitudinal data to track the effects of different genotypes of SAP97 rs3915512 on the progression of disease and response to drugs.

## Data Availability Statement

The datasets presented in this study can be found in online repositories. The names of the repository/repositories and accession numbers can be found in the article/Supplementary Material.

## Ethics Statement

The studies involving human participants were reviewed and approved by the Ethics Committee of the Affiliated Hospital of Guangdong Medical University. The patients/participants provided their written informed consent to participate in this study.

## Author Contributions

ZLu, YW, and GM conceived and designed the experiments and revised the manuscript. XWa, JY, and XL did genetic analyses. BH, SX, DZ, CL, and JF collected the clinical data. ZLi, DL, and ZD collected the imaging data. JL, YL, and WC analyzed and interpreted the data. XX, SL, and XWe drafted the manuscript. All authors contributed to the article and approved the submitted version.

## Conflict of Interest

The authors declare that the research was conducted in the absence of any commercial or financial relationships that could be construed as a potential conflict of interest.
