# Supplementary material for: Genetic Contribution of Synapse-Associated Protein 97 to Orbitofrontal–Striatal–Thalamic Circuitry Connectivity Changes in First-Episode Schizophrenia
Source: Front Psychiatry. 2021 Jul 19;12:691007. doi: 10.3389/fpsyt.2021.691007 (PMC8326367; doi:10.3389/fpsyt.2021.691007)
Supplement: Supplementary file 1 [file Table_1.DOC]

**Genetic contribution of synapse-associated protein 97 to orbitofrontal-striatal-thalamic circuitry connectivity changes in first-episode schizophrenia**

Xusan Xu^1, 2#^, Shucun Luo^3#^, Xia Wen^1#^, Xiaoxia Wang^1^, Jingwen Yin^4^, Xudong Luo^4^, Bin He^3^ Chunmei Liang^1^, Susu Xiong^4^, Dongjian Zhu^4^, Jiawu Fu^1^, Dong Lv ^4^, Zhun Dai^4^, Juda Lin^4^, You Li^1^, Zhixiong Lin^4^, Wubiao Chen^3^, Zebin Luo^3*^, Yajun Wang^5*^, Guoda Ma^1,2*^

1. Institute of Neurology, Affiliated Hospital of Guangdong Medical University, Zhanjiang, 524001, China

2. Maternal and Children's Health Research Institute, Shunde Women and Children’s Hospital, Guangdong Medical University, Foshan, 528300, China

3. Department of Radiology, Affiliated Hospital of Guangdong Medical University, Zhanjiang, 524001, China

4. Department of Psychiatry, Affiliated Hospital of Guangdong Medical University, Zhanjiang, 524001, China

5. Clinical Research Center, Affiliated Hospital of Guangdong Medical University, Zhanjiang, 524001, China

**Subjects**

One hundred and four subjects, including 52 schizophrenic patients (30 males and 22 females) and 52 healthy controls (23 males and 29 females), were recruited in this study. All participants were recruited at the Health Examination Center and the Department of Psychiatry of the Affiliated Hospital of Guangdong Medical University from July 2014 to August 2019. Both groups were matched for sex, age and education level. The study was approved by the ethics committee of the Affiliated Hospital of Guangdong Medical University.

These subjects were all unrelated southern Han Chinese who met the diagnostic criteria of schizophrenia in the Diagnostic and Statistical Manual of Mental Disorders V (DSM-V) assessed by at least two well-trained senior psychiatrists (2010). In this study, patients had no history of organic brain damage, metabolic diseases, organic mental disorders or autoimmune diseases and controls had no family history of mental illness (first-degree relatives) or substance abuse. Demographic information on the subjects was collected, including age, nationality, gender, family history, education, age of onset and the disease course.

At least two trained clinical neurologists conducted interviews with schizophrenic patients to rate symptom severity and cognitive functions using the Positive and Negative Symptom Scale (PANSS) (Nicotra et al., 2015) and the Brief Assessment of Cognition in Schizophrenia (BACS) scale (Keefe et al., 2004).

**Genotyping**

Genomic DNA was extracted following the instructions provided by a whole-blood DNA extraction kit (Tiangen Biotech, Beijing, China). Then, genotyping all 104 subjects for rs3915512 used the improved multiplex ligation detection reaction (imLDR) technique (Genesky Biotech, Shanghai, China) described in our previous study (Xu et al., 2020).

# Imaging data acquisition

Images were collected in a 3.0 T GE Discovery MR750 scanner (GE Healthcare Systems, Milwaukee, WI, USA) system with an 8-channel head coil. Subjects were asked to close their eyes, stay awake, keep still and minimize mental activity during the scan.

T1-weighted data acquisition was obtained via a three-dimensional (3D) fast field echo (FFE) pulse sequence (major parameters: echo time (TE) = 3.18 ms, repetition time (TR) = 8.16 ms, flip angle (FA): 90°, scanning slice: 172 slices, slice thickness: 1 mm, slice interval: 0 mm, field of view (FOV) = 512 mm × 512 mm, matrix: 256 × 256).

The fMRI data were obtained through an echo planar imaging (EPI) sequence with the following parameters: TE = 30 ms, TR = 2000 ms, FA: 90°, scanning slice: 38 slices, slice thickness: 3.6 mm, slice interval: 0.6 mm, FOV = 230 mm × 230 mm, matrix: 64 × 64, scanning time: 8 min, and 240 dynamic scans.

Diffusion tensor imaging (DTI) data acquisition was collected using spin echo-echo plane imaging (SE EPI) with TE= 85 ms, TR= 12000 ms, scanning slice: 75 slices, slice thickness: 2.0 mm, slice interval: 0.6 mm, FOV = 230 mm × 230 mm, matrix: 128 × 128, b = 1000 s/mm2, 32 gradient directions and one scan without diffusion gradient (b = 0 s/mm2), and scanning time: 7 min and 12 seconds.

## Data processing

## The analysis of the fMRI data was performed by a data processing assistant for resting-state fMRI software (DPARSF_V2.3, Cognitive and Brain Diseases Centre of Hangzhou Normal University), which was implemented in MATLAB 2012a.

The main steps for fMRI data processing included (1) converting files from the DICOM format into the NIfTI format; (2) discarding the first 10 time points for each subject; (3) correcting for slice timing; (4) correcting for rigid-body head motion (rotational or translational motion parameters less than 2.5° or 2.5 mm); (5) normalizing to the MNI template space and resampling with 3 mm × 3 mm × 3 mm voxels; (6) spatial smoothing (6-mm full-width at half-maximum (FWHM) Gaussian kernel); (7) filtering (0.01–0.08 Hz); and (8) regressing out nuisance signals including head motion parameters by using the Friston 24 model, global signal, cerebrospinal fluid signal and white matter signal. (9) Seed-based region of interest (ROI) functional connectivity analysis was performed by placing seeds with 16 MNI coordinates in the orbitofrontal gyrus, striatum and thalamus defined by previous researchers (Table S1) (Power et al., 2011). The time series signals around these coordinates with a radius of 6 mm were extracted, and the connections within these 16 functional nodes were analysed. For the individual seed connectivity map, Fisher's r-to-z transformation was used.

The analysis of the DTI data was performed by the software pipeline toolbox for analysing brain diffusion images (PANDA, State Key Laboratory of Cognitive Neuroscience and Learning and IDG/McGovern Institute for Brain Research, Beijing Normal University) (Cui et al., 2013).

Major steps for DTI data processing included (1) converting files from the DICOM Format into the NIfTI Format; (2) extracting the brain and skull removal= 0.25; (3) cropping the image; (4) correcting eddy current/motion; (5) averaging acquisitions; (6) registering individual fractional anisotropy (FA) images of native space to the FA template in the Montreal Neurological Institute (MNI) space and then applying the resultant warping transformations to write the images of the diffusion metrics into the MNI space; (7) implementing deterministic tracking using the deterministic fibre tracking algorithm Fibre Assignment by Continuous Tracking (FACT), which stopped when the line encountered a track with a turning angle of more than 45° (angle threshold) or a voxel with an FA value of less than 0.2 or more than 1.0 (FA threshold). Based on whole-brain fibres, three weighted matrices (fibre number matrix, average FA weighted matrix and average fibre length matrix) were acquired.

**References**

2010. Psychiatry. DSM-V at a glance. *Science,* 327**,** 770-1.

CUI, Z., ZHONG, S., XU, P., HE, Y. & GONG, G. 2013. PANDA: a pipeline toolbox for analyzing brain diffusion images. *Front Hum Neurosci,* 7**,** 42.

KEEFE, R. S., GOLDBERG, T. E., HARVEY, P. D., GOLD, J. M., POE, M. P. & COUGHENOUR, L. 2004. The Brief Assessment of Cognition in Schizophrenia: reliability, sensitivity, and comparison with a standard neurocognitive battery. *Schizophr Res,* 68**,** 283-97.

NICOTRA, E., CASU, G., PIRAS, S. & MARCHESE, G. 2015. On the use of the Positive and Negative Syndrome Scale in randomized clinical trials. *Schizophr Res,* 165**,** 181-7.

POWER, J. D., COHEN, A. L., NELSON, S. M., WIG, G. S., BARNES, K. A., CHURCH, J. A., VOGEL, A. C., LAUMANN, T. O., MIEZIN, F. M., SCHLAGGAR, B. L. & PETERSEN, S. E. 2011. Functional network organization of the human brain. *Neuron,* 72**,** 665-78.

XU, X., WANG, Y., ZHOU, X., YIN, J., YU, H., WEN, X., LV, D., ZHU, D., XIONG, S., YAN, H., DAI, Z., LIN, Z., LIN, J., ZHAO, B., LIANG, C., LI, Y., LUO, X. & MA, G. 2020. The genetic variations in SAP97 gene and the risk of schizophrenia in the Chinese Han population: a further study. *Psychiatr Genet,* 30**,** 110-118.

| Table S1. 16 MNI spatial coordinates of involved brain area. | | | |
| --- | --- | --- | --- |
| AAL | MNI | AAL | MNI |
| left superior frontal gyrus, orbital part | -18,63,-9 | left caudate | -17,14,16 |
| right superior frontal gyrus, orbital part | 24,45,-15 | right caudate | 14,-4,22 |
| left middle frontal gyrus, orbital part | -42,45,-2 | left putamen | -22,7,-5 |
| right middle frontal gyrus, orbital part | 34,54,-13 | right putamen | 31,-14,2 |
| left inferior frontal gyrus, orbital part | -46,31,-13 | left pallidum | -15,4,8 |
| right inferior frontal gyrus, orbital part | 49,35,-12 | right pallidum | 15,5,7 |
| left superior frontal gyrus, medial orbital | -3,44,-9 | left thalamus | -10,-18,7 |
| right superior frontal gyrus, medial orbital | 8,42,-5 | right thalamus | 9,-4,6 |
| AAL: Anatomical Automatic Labeling; MNI: Montreal Neurological Institute. | | | |

| Table S2. Interactive effect and post hoc analysis of RSFC values between SAP97 rs3915512 genotype and disease. | | | | | | | | | |
| --- | --- | --- | --- | --- | --- | --- | --- | --- | --- |
|  | | RSFC value | | | |  | | post hoc analysis of genotype in FES | |
| AAL | | HC | | FES | | Interactive effect | |
|  |  | TT | TA+AA | TT | TA+AA | *F* | *P* | *F* | *P* |
| ORBsup-R | Thalamus-L | -0.02±0.15 | -0.25±0.27 | -0.03±0.27 | 0.08±0.26 | 13.50 | 3.92E-04 | 3.16 | 0.078 |
| ORBsup-R | Pallidum-R | 0.16±0.13 | 0±0.20 | 0.12±0.26 | 0.24±0.23 | 10.98 | 0.001 | 4.49 | 0.037 |
| ORBmid-R | Putamen-L | 0.14±0.18 | 0.02±0.19 | 0.05±0.22 | 0.25±0.22 | 14.20 | 2.83E-04 | 11.09 | **0.001** |
| ORBmid-R | Putamen-R | 0.15±0.22 | 0.03±0.25 | 0.03±0.26 | 0.22±0.19 | 11.28 | 0.001 | 8.65 | **0.004** |
| ORBsupmed-L | Thalamus-L | 0.11±0.20 | -0.03±0.25 | -0.02±0.18 | 0.19±0.27 | 16.23 | 1.12E-04 | 11.89 | **0.001** |
| ORBsupmed-R | Thalamus-L | 0.11±0.18 | -0.05±0.29 | 0.03±0.24 | 0.20±0.21 | 13.59 | 3.76E-04 | 7.23 | **0.008** |
| AAL: Anatomical Automatic Labeling; HC: healthy control; FES: first episode schizophrenia; values are the mean ± SD; 2 × 2 ANCOVA P< 0.00625; The bold values in the post hoc analysis can survive for Bonferroni correction (P< 0.0125). | | | | | | | | | |
|
| R: right; L: left; ORBsup: superior frontal gyrus, orbital part; ORBmid: middle frontal gyrus, orbital part; ORBsupmed: superior frontal gyrus, medial orbital. | | | | | | | | | |
|

| Raw data of this study | | | | |
| --- | --- | --- | --- | --- |
| Sample Name | Gender | Age (year) | Years of Education | rs3915512 |
| FES1 | female | 36 | 6 | T/T |
| FES2 | female | 16 | 9 | T/T |
| FES3 | male | 40 | 12 | T/T |
| FES4 | male | 23 | 12 | T/T |
| FES5 | female | 26 | 16 | T/T |
| FES6 | female | 19 | 12 | T/T |
| FES7 | male | 23 | 9 | T/T |
| FES8 | male | 32 | 9 | T/T |
| FES9 | female | 34 | 9 | T/T |
| FES10 | male | 25 | 12 | T/T |
| FES11 | female | 49 | 12 | T/T |
| FES12 | female | 21 | 9 | T/T |
| FES13 | female | 19 | 12 | T/T |
| FES14 | female | 22 | 12 | T/T |
| FES15 | female | 25 | 7 | T/T |
| FES16 | male | 17 | 9 | T/T |
| FES17 | male | 30 | 9 | T/T |
| FES18 | male | 31 | 16 | T/T |
| FES19 | male | 21 | 12 | T/T |
| FES20 | male | 24 | 9 | T/T |
| FES21 | female | 29 | 9 | T/T |
| FES22 | female | 31 | 8 | T/T |
| FES23 | male | 28 | 6 | T/T |
| FES24 | male | 26 | 7 | T/T |
| FES25 | female | 16 | 10 | T/T |
| FES26 | male | 20 | 15 | T/T |
| FES27 | male | 28 | 9 | T/T |
| FES28 | male | 21 | 13 | T/T |
| FES29 | male | 40 | 9 | T/A |
| FES30 | male | 25 | 12 | T/A |
| FES31 | male | 25 | 12 | T/A |
| FES32 | male | 23 | 15 | T/A |
| FES33 | female | 30 | 9 | T/A |
| FES34 | female | 20 | 12 | T/A |
| FES35 | female | 18 | 9 | T/A |
| FES36 | female | 25 | 6 | T/A |
| FES37 | male | 20 | 15 | T/A |
| FES38 | male | 47 | 12 | T/A |
| FES39 | male | 43 | 9 | T/A |
| FES40 | male | 25 | 9 | T/A |
| FES41 | female | 42 | 9 | T/A |
| FES42 | male | 22 | 9 | T/A |
| FES43 | female | 37 | 9 | T/A |
| FES44 | male | 30 | 9 | T/A |
| FES45 | female | 22 | 7 | T/A |
| FES46 | male | 27 | 16 | T/A |
| FES47 | female | 42 | 16 | T/A |
| FES48 | female | 24 | 9 | T/A |
| FES49 | male | 36 | 12 | T/A |
| FES50 | male | 17 | 12 | T/A |
| FES51 | male | 26 | 12 | A/A |
| FES52 | male | 21 | 12 | A/A |
| HC1 | male | 21 | 14 | T/T |
| HC2 | female | 21 | 14 | T/T |
| HC3 | male | 21 | 14 | T/T |
| HC4 | male | 22 | 15 | T/T |
| HC5 | female | 22 | 15 | T/T |
| HC6 | female | 22 | 15 | T/T |
| HC7 | male | 21 | 14 | T/T |
| HC8 | female | 22 | 15 | T/T |
| HC9 | female | 21 | 14 | T/T |
| HC10 | female | 36 | 6 | T/T |
| HC11 | female | 32 | 9 | T/T |
| HC12 | male | 49 | 9 | T/T |
| HC13 | male | 46 | 9 | T/T |
| HC14 | female | 45 | 9 | T/T |
| HC15 | male | 40 | 12 | T/T |
| HC16 | male | 45 | 12 | T/T |
| HC17 | male | 31 | 9 | T/T |
| HC18 | male | 41 | 12 | T/T |
| HC19 | female | 32 | 9 | T/T |
| HC20 | female | 38 | 9 | T/T |
| HC21 | male | 32 | 15 | T/T |
| HC22 | female | 24 | 15 | T/T |
| HC23 | female | 26 | 15 | T/T |
| HC24 | male | 28 | 5 | T/T |
| HC25 | female | 27 | 12 | T/T |
| HC26 | male | 21 | 9 | T/T |
| HC27 | male | 26 | 12 | T/T |
| HC28 | female | 21 | 12 | T/T |
| HC29 | female | 28 | 12 | T/T |
| HC30 | female | 23 | 12 | T/T |
| HC31 | female | 22 | 15 | T/A |
| HC32 | female | 21 | 14 | T/A |
| HC33 | female | 20 | 13 | T/A |
| HC34 | female | 38 | 9 | T/A |
| HC35 | female | 32 | 9 | T/A |
| HC36 | female | 42 | 9 | T/A |
| HC37 | female | 24 | 12 | T/A |
| HC38 | male | 37 | 12 | T/A |
| HC39 | male | 38 | 12 | T/A |
| HC40 | male | 21 | 12 | T/A |
| HC41 | male | 36 | 12 | T/A |
| HC42 | female | 32 | 12 | T/A |
| HC43 | female | 45 | 6 | T/A |
| HC44 | female | 22 | 15 | T/A |
| HC45 | female | 20 | 12 | T/A |
| HC46 | female | 22 | 12 | T/A |
| HC47 | male | 26 | 12 | T/A |
| HC48 | male | 32 | 9 | T/A |
| HC49 | male | 32 | 9 | T/A |
| HC50 | female | 24 | 9 | A/A |
| HC51 | male | 20 | 13 | A/A |
| HC52 | male | 27 | 12 | A/A |
| HC: healthy control; FES: first episode schizophrenia. | | | | |
